# Evolution of a unique predatory feeding apparatus: functional anatomy, development and a genetic locus for jaw laterality in Lake Tanganyika scale-eating cichlids

**DOI:** 10.1186/1741-7007-8-8

**Published:** 2010-01-26

**Authors:** Thomas A Stewart, R Craig Albertson

**Affiliations:** 1 Department of Biology, Syracuse University, 130 College Place, Syracuse, NY, 13244, USA

## Abstract

**Background:**

While bilaterality is a defining characteristic of triploblastic animals, several assemblages have managed to break this symmetry in order to exploit the adaptive peaks garnered through the lateralization of behaviour or morphology. One striking example of an evolved asymmetry in vertebrates comes from a group of scale-eating cichlid fishes from Lake Tanganyika. Members of the Perissodini tribe of cichlid fishes have evolved dental and craniofacial asymmetries in order to more effectively remove scales from the left or right flanks of prey. Here we examine the evolution and development of craniofacial morphology and laterality among Lake Tanganyika scale-eating cichlids.

**Results:**

Using both geometric and traditional morphometric methods we found that the craniofacial evolution in the Perissodini involved discrete shifts in skeletal anatomy that reflect differences in habitat preference and predation strategies. Further, we show that the evolutionary history of the Perissodini is characterized by an accentuation of craniofacial laterality such that certain taxa show elaborate sided differences in craniofacial shape consistent with the sub-partitioning of function between sides of the head during attacks. Craniofacial laterality in the scale-eating specialist *Perissodus microlepis *was found to be evident early in development and exhibited a unimodal distribution, which is contrary to the adult condition where jaw laterality has been described as a discrete, bimodal antisymmetry. Finally, using linkage and association analyses we identified a conserved locus for jaw handedness that segregates among East African cichlids.

**Conclusions:**

We suggest that, during the evolution of the Perissodini, selection has accentuated a latent, genetically determined handedness of the craniofacial skeleton, enabling the evolution of jaw asymmetries in order to increase predation success. Continued work on the developmental genetic basis of laterality in the Perissodini will facilitate a better understanding of the evolution of this unique group of fishes, as well as of left-right axis determination among vertebrates in general.

## Background

Most multicellular animals exhibit one of two forms of symmetry: radial, in which multiple planes of symmetry can be drawn across an organism; or bilateral, where a single plane of symmetry, the sagittal plane, bisects an organism into mirrored halves [1]. Bilateral symmetry is a synapomorphy of the bilateria, a taxonomic group that encompasses most animal phyla. Despite its utility as a diagnostic character, however, symmetry is not ubiquitous across all organ systems. For example, there is marked asymmetry in the patterning of the brain, heart and visceral organs in vertebrates and the genes that regulate the asymmetric morphogenesis of these structures (for example, *nodal, lft1, pitx2*) are well known [2]. Likewise, a myriad of asymmetries have evolved in normally paired structures among various vertebrate lineages. Owls have evolved asymmetrical ears, which differ in size and placement on the skull, making them more effective auditory predators [3]. The eyes of flatfish migrate over the midline of the body during development and the oral jaws develop asymmetrically such that adults can lie on the benthic substrate and ambush prey [4]. Conspicuous craniofacial asymmetries are also evident in narwals [5], fruit bats [6] and a group of snail-eating snakes [7]. The prevalence of laterality in nature suggests that bilateral symmetry may, in fact, be more superficial than originally thought and, while much is known about the developmental genetic basis for normal asymmetric development of the visceral organs and brain, comparatively little is known about the genetic basis of laterality in normally paired structures.

Asymmetries are typically differentiated according to their causal origin and tend to be grouped into three classes. The first is fluctuating asymmetry, where the breaking of symmetry is a consequence of developmental 'noise' and lacks a strict genetic basis. Asymmetries of this type are normally distributed around a mean symmetrical form [8]. The second type of asymmetry is antisymmetry, where the nature of the asymmetry (that is, which traits are affected) is genetically determined, but the side in which the trait manifests itself is purported to be environmentally determined [9,10]. The random environmental determination of handedness results in an equal bimodal distribution on either side of a symmetrical mean [10]. The third category is directional asymmetry, in which both the trait of interest and handedness are genetically determined. Directional asymmetries are found in populations as skewed unimodal distributions, with populations biased towards a particular side. The evolution of directional asymmetries is thought to occur either directly from a symmetrical ancestor or as a progression from symmetry to antisymmetry to directional asymmetry [9,11].

Scale-eating cichlids from Lake Tanganyika present a striking example of an evolved asymmetry in mouth direction. The Perissodini are a monophyletic cichlid tribe from Lake Tanganyika whose evolutionary history is marked by an ecological expansion from a deep-water generalized predator to shallow-water specialists that feed almost exclusively on scales (lepidophagy) [12]. Within the Perissodini, *Perissodus *species exhibit jaw asymmetries that are dimorphic [13-18], with mixed populations of both 'lefty' individuals, that attack the left side of prey species with mouths angled off to the right, and 'righty' individuals, that correspondingly attack the right side with mouths bent to the left [19]. The nature of this asymmetry has been attributed to sided differences in the length of the jaw joint (that is, the left-side is longer in lefty individuals) [15] but little more detail has been offered.

In *P. microlepis *lefty and righty morphs are maintained through frequency-dependent selection, where the minority morph experiences a higher fitness than the majority morph as a consequence of a preferential prey avoidance of the more abundant morph [14]. The relative frequency of each morph fluctuates around a mean of 0.5 and the presence of both morphs appears to be an evolutionary stable state [14]. This system is a commonly cited example of antisymmetry, as jaw asymmetry is bimodal and there appears to be no species level bias in handedness [10]. Although the handedness of antisymmetric traits is generally assumed to be environmentally determined, a genetic basis has been suggested for jaw laterality in *P. microlepis *[14,19], as well as for the freshwater goby *Rhinogobius flumineus *[20] and the herbivorous cichlid *Neolamprologus moorii *[19], suggesting widespread heritable laterality in mouth direction among Perciformes. Jaw laterality, therefore, does not appear to fit neatly into any one category of asymmetry [10]. The bimodal distribution of mouth direction in *P. microlepis *populations is what would be expected for an antisymmetric trait [13]. However, a genetic basis for jaw handedness is more consistent with a directional asymmetry [10]. This apparent paradox has led to some debate in the literature concerning the causal origin of this trait [10].

Here we explore the evolution of craniofacial morphology and laterality among scale-eating cichlids. Using geometric and traditional morphometric techniques, we show that the evolution of the Perissodini involved discrete shifts in craniofacial shape that are correlated with foraging habitat and that sided differences in craniofacial anatomy are evident in certain species that feed exclusively on scales, consistent with the lateralization of feeding mechanics. In *P. microlepis*, we observe jaw laterality early in development and identify a conserved locus segregating with craniofacial handedness in East African cichlids. Our data are consistent with the hypothesis that jaw laterality evolved in Tanganyikan scale-eaters due to selection on a conserved locus for handedness and that, as species became increasingly specialized to feed on scales, selection favoured an elaboration of this asymmetry through the evolution of sided differences in jaw shape.

## Results and discussion

### Shifts in craniofacial anatomy correspond to different foraging niches

**Figure 1:**
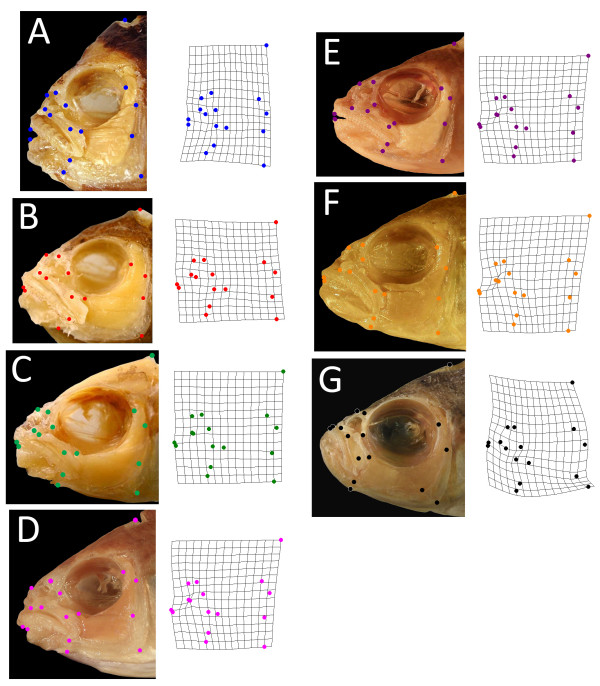
**Representative individuals of Perissodini species included in shape analyses**. (A) *Perissodus straeleni*; (B) *P. microlepis*; (C) *P. paradoxus*; (D) *P. elaviae*; (E) *P. multidentatus*; (F) *P. hecqui*; (G) *Haplotaxodon microlepis*. Both the left and right sides of the head were photographed and analysed for all species except *H. microlepis *for which only the left-hand side was examined, as this species does not exhibit an overt jaw asymmetry. Landmarks represent functionally significant points that characterize the geometry of the skull and were adapted from Cooper and Westneat [34]. Images are of representative Perissodini species with head shapes that are close to the species mean for craniofacial shape (that is, at the centre of each species' two-dimensional distribution shown in Figure 2). Corresponding warps represent the average two-dimensional head shape (including both the left and right sides of the head) in each species.

**Figure 2:**
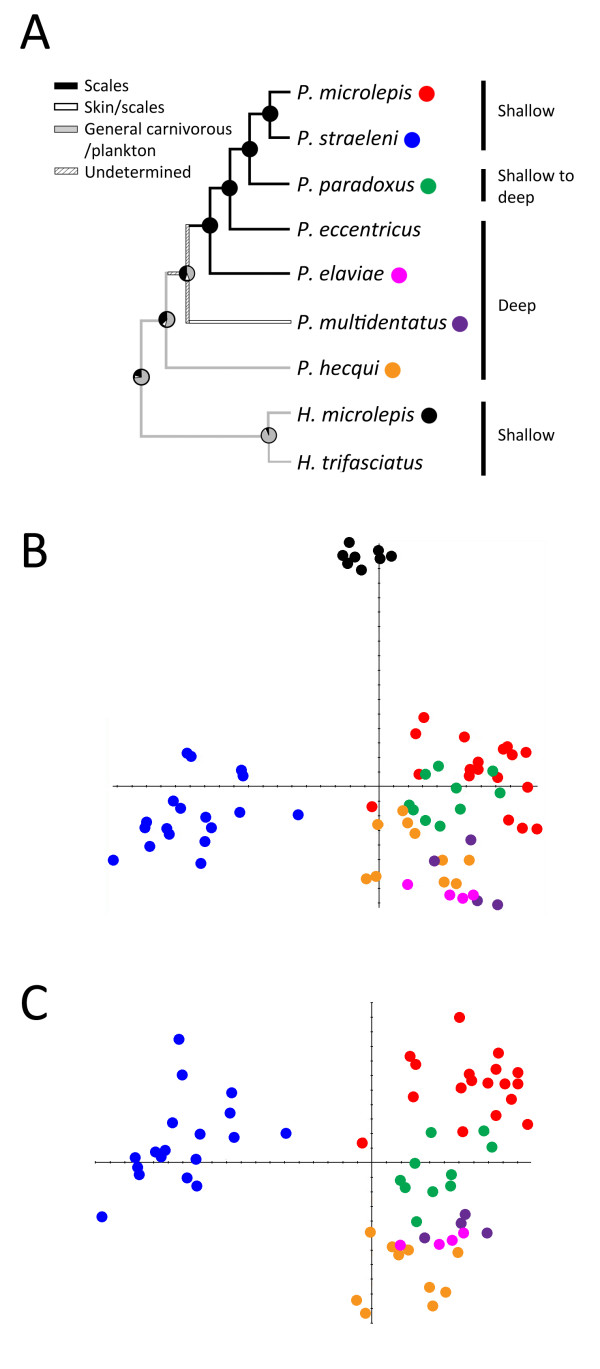
**Phylogenetic history and craniofacial morphospace of the Perissodini**. (A) Ancestral state reconstruction for foraging habits and diet of the Perissodini, modified from Takahashi *et al.*[12]. Ancestral foraging preferences are labelled on the tree and preferred habitat depths are indicated to the right. Species are colour coded according to the scatter plots in B and C. Relative warp (RW) analysis revealed several shifts in craniofacial anatomy during Perissodini diversification that correlate with shifts in ecological foraging niches (B, C). (B) Among the Perissodini, RW1 accounts for 39.87% of the variation in shape among species and represents variation in skull length. RW2 accounts for 24.22% of shape variation among species and reflects differences among species in the angle of the mouth. (C) When considering only those species in the genus *Perissodus*, RW1 accounts for 50.88% of the variation in shape among species and represents variation in skull length. RW2 reflects variation in eye size and accounts for 17.00% of the shape variation.

Relative warp (RW) analysis was performed in order to compare the craniofacial architecture of seven species within the Perissodini tribe (Figures 1 and 2). RW1 accounted for 40% of total shape variation among individuals and described variation in skull length. RW2 accounted for 24% of shape variation and reflected differences in the rotation of the mouth (Figure 2B). *Haplotaxodon microlepis *has a dramatically up-turned mouth and is significantly different from all other species along RW2 (Figure 1G). A second relative warp analysis was performed excluding *H. microlepis *in order to better resolve shape variation among species in the genus *Perissodus *(Figure 2C). As with the previous analysis, RW1 (now 51%) described shifts in skull length but RW2 (17%) characterized differences in eye size (Figure 2C).

These shifts in anatomy correspond to differences in foraging niche. *H. microlepis *is a member of the Perissodini tribe, and is a sister taxon to the *Perissodus *genus (Figure 2A). The only symmetrical species among those analysed, it is a shallow water predator with large eyes and an up-turned mouth. Much of its diet is composed of small, pelagic *Mysis *shrimps and juvenile fishes [12,21]. *Mysis *shrimps are extremely light sensitive and tend to be nocturnal [22,23]. Large eyes, similar to those of deep-water species, would facilitate predation on this food source. *H. microlepis *also feeds on schools of juvenile fish that occupy the upper few centimetres of the water column and it has been proposed that its up-turned mouth allows it to exploit this resource by stalking and attacking prey from below [21]. Among the *Perissodus *species, scale-eating evolved from a more generalized predation strategy in deep water carnivores [12], a trend that is reflected in the large eye size of *Perissodus *species that still forage at depth (Figures 1 and 2). Among the *Perissodus *species that forage in the shallows, a divergence in skull length is observed (Figure 2). The sister taxa, *P. microlepis *and *P. straeleni*, lay at opposite ends of this axis and exhibit pronounced differences in head length (Figure 2), which probably reflect differences in predatory behaviour [16]. The short skull length and deep body of *P. straeleni *allows it to attack from short distances, relying on manoeuverability to capture prey, while the longer skulls and shallow bodies of *P. microlepis *facilitate attacks from greater distances, employing speed to successfully capture prey [16]. These strategies represent alternate adaptations for lepidophagy in shallow water with high visibility [16].

### Scale eaters exhibit sided differences in craniofacial shape

**Figure 3:**
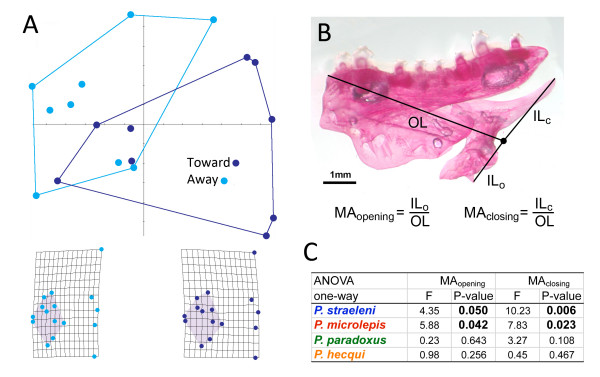
***Perissodus *species exhibit sided differences in craniofacial shape**. (A) *Perissodus straeleni *shows marked differences in the entire geometry of the skull associated with a general lengthening of the preorbital region (shaded region of the grids) of the skull on the side facing prey. (B) Lever models of the lower jaw were used in order to evaluate the functional implications of jaw asymmetry. (C) Analysis of variance, comparing the mechanical advantage of opening and closing lever systems in the lower jaw, predicts sided differences in the force and speed of the lower jaw in both *P. straeleni *and *P. microlepis*.

Asymmetries in the length of the jaw joint and mouth orientation were noted for all *Perissodus *species, consistent with previous descriptions [13-15,17,18]. Subtle, but consistent, asymmetries were also observed for a variety of traits, including the thickness of the maxilla and premaxilla and curvature of the nasal bone, underscoring the complexity of this phenotype. We next wanted to test whether *Perissodus *species exhibited quantitative asymmetries in craniofacial shape. Using relative warp analysis to compare the 'towards' (facing prey) and 'away' (opposite to prey) sides within species, we found that only *P. straeleni*, a shallow water scale-specialist, exhibited an asymmetry in overall head shape (Figure 3A). Sided differences in this species were characterized by variation in the length of the preorbital region of the skull (grids, Figure 3A). These data suggest an integration of effects across broad regions of the head, with an overall elongation of the preorbital region of the skull noted for the side of the head facing prey during attacks.

Next, we tested whether *Perissodus *species exhibited consistent asymmetries in specific functionally relevant characters of the jaw. In order to do this we modelled the mechanics of the lower jaw as a first order lever, where the fulcrum was taken as the jaw joint, the out-lever was measured as the distance between the foremost tip of the jaw and the fulcrum, the opening in-lever was estimated as the distance from the tip of the retroarticular process of the lower jaw to the fulcrum and the closing in-lever was the distance from the end of the ascending arm of the lower jaw to the fulcrum [24] (Figure 3). From these lengths, mechanical advantage (MA) was calculated for jaw opening and closing as the ratio of in-lever to out-lever lengths, which can be used to estimate the relative speed and force of a lever [24]. A high MA is predictive of fish with powerful bites, while a low MA is predictive of fast, weaker bites [25]. We found that both *P. straeleni *and *P. microlepis *exhibited discrete sided differences in the MA of both the jaw opening and closing lever systems, predicting a lateralization in the speed and force of lower jaw rotation (Figure 3B and 3C). For both species, relatively lower MAs were estimated for the side of the fish that would be closest to the prey (toward the prey), consistent with a coordinated partitioning of force and speed between different sides of the jaw. No biases in MA were detected in other *Perissodus *species, which suggests that asymmetries have been elaborated in species adapted for shallow-water foraging strategies to include functionally significant sided differences in jaw shape. The exact nature and biological relevance of these functional asymmetries remain to be explored. One reasonable hypothesis is that, as the *Perissodus *foraging niche expanded from deep-water generalized predation to shallow-water specialization on scale-eating [12], selection favoured modifications of the feeding apparatus in order to increase foraging success, including the sub-partitioning of feeding mechanics between opposite sides of the jaw.

### Jaw laterality is evident early in development and is not distributed as an antisymmetric trait

**Figure 4:**
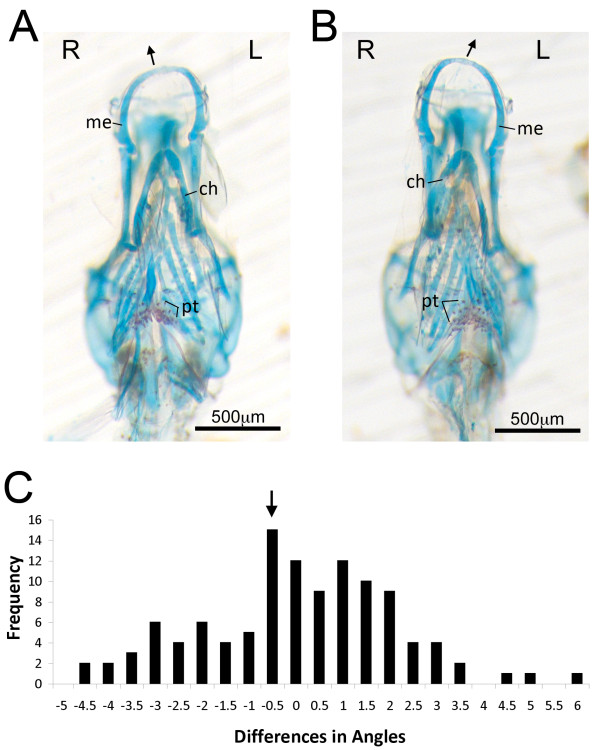
**Larval *Perissodus microlepis *individuals exhibit discrete pharyngeal skeletal asymmetries**. A clutch of *P. microlepis *was collected from the mouth of a wild-caught female and staged to the late larval period. Skeletal preparations revealed clear asymmetries in a subset of these individuals (A, B), including sided differences in the shape of Meckel's (me) and ceratohyal (ch) cartilages. The degree of asymmetry for each individual was quantified following Hori *et al. *[19], in which the differences in the angle from the preorbital processes to the premaxillary symphysis was determined. Unlike that found for adult fish, the distribution of larval asymmetries is unimodal (C). The arrow indicates mean laterality for the clutch. Abbreviation: pt, pharyngeal teeth.

A brood of 141 larval *P. microlepis *were collected from the mouth of a lefty female, and were staged to the late larval period [26]. Each animal retained a significant amount of yolk, indicating that they had not yet begun to feed for themselves, and the pharyngeal skeleton had only just begun to mineralize. Nevertheless, conspicuous craniofacial asymmetries were observed in many of these larval samples (Figure 4A and 4B), consistent with a genetic basis for jaw handedness. Asymmetries were noted for jaw direction, hyoid length and curvature and pharyngeal jaw dentition. The degree of asymmetry varied widely among individuals and, while both lefty and righty individuals were clearly present, the distribution of jaw laterality was unimodal, suggesting that this textbook antisymmetric trait has a more complicated developmental origin.

The distribution of larval jaw asymmetry was biased to the right-side (lefty). However, the family-level mean was not statistically different from zero (one-sample *t*-test, *t *= -0.87, *P *= 0.39) (Figure 4C). While a genetic basis for jaw laterality among *P. microlepis *would make the most sense in the context of a directional asymmetry, it will require the analysis of additional individuals from multiple families to determine whether this early larval phenotype represents a fluctuating or directional asymmetry.

The observation of an early unimodal distribution of jaw asymmetry in scale eaters could provide a solution to the *Perissodus *paradox. However, it would require a population-level shift from a unimodal distribution in young fish to a discrete, bimodal antisymmetry in adults. Whether, and how, such a shift might occur would be a fruitful topic for future investigations. It is possible that the early asymmetry in jaw patterning could arise through the co-option of the conserved asymmetric signalling pathway used to define the left-right axis in vertebrates. Work on zebrafish has demonstrated that modulating distinct members of this signaling cassette not only disrupts normal asymmetric patterning, but also breaks symmetric patterning of paired structures, including the pharyngeal skeleton and somites [27-29]. Moreover, these mutational effects are of a directional (not fluctuating) nature, affecting one side of the body more often than the other, which suggests that they are not the result of developmental noise. Once this unimodal asymmetry has been established in young fishes, the transition to antisymmetry could involve asymmetric growth and remodelling of the jaw [15], leading to a shift away from the mean and an accentuation of the tails of the distribution. Haploinsufficiency of zebrafish *fgf8 *has been linked to asymmetric remodelling of the craniofacial skeleton, offering a putative molecular mechanism for this phenomenon [28]. The lateralization of foraging behaviour might also influence the transition to antisymmetry. Lateralizations in behaviour have been well studied in vertebrates and relationships between behavioural and morphological lateralization have been documented for many fish species. A behavioural laterality in zebrafish swimming, for example, has been associated with sided differences in white and red muscle masses [30] and asymmetric foraging behaviours have been linked to mouth laterality in a number of species [13,14,20,31]. In *P. microlepis*, an early directional asymmetry might be accentuated and reinforced by lateralized foraging behaviour and jaw plasticity through a positive feedback loop, wherein the behavioural asymmetry would lead to the asymmetric remodelling of the jaw (which would be most pronounced in near symmetric larval fish). This would lead to a further functional lateralization and increased foraging success which, in turn, would reinforce the behavioural asymmetry, and so on. Finally, it is likely that selection has shaped the bimodal distribution of jaw handedness observed in adult *P. microlepis*. If symmetric, or nearly symmetric, fry were selected against and those with asymmetric jaws were selected for, this would lead to a population-level shift from a unimodal distribution in juveniles to a bimodal distribution in adults. In all likelihood, a confluence of factors underlie the development of jaw handedness in scale-eating cichlids, leaving many questions to be answered regarding (i) the genetic basis, (ii) ontogeny and (iii) plasticity of jaw laterality in the Perissodini.

### Identification of a conserved locus for jaw handedness

**Figure 5:**
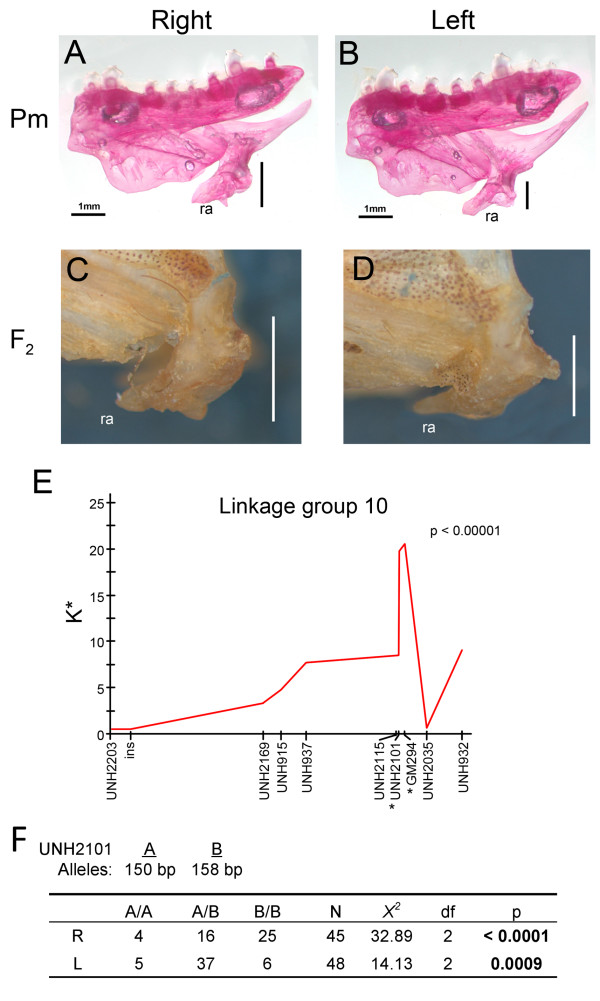
**Identification of a conserved locus for jaw laterality**. A prominent feature of jaw laterality in *Perissodus microlepis *is a sided difference in the length of the retroarticular processes (ra) of the jaw (A, B). We found that a similar asymmetry segregates in the F_2 _from an interspecific cross between two herbivorous species from Lake Malawi (C, D). (E) Linkage analysis revealed a significant association between handedness of retroarticular length and two linked markers on linkage group 10. (F) Larval *P. microlepis *were typed as either righty (R) or lefty (L) and genotyped at both markers. GM294 was invariant, but UNH2101 showed a size polymorphism that segregated with handedness in this family. Patterns of inheritance are consistent with dominance of the lefty allele and early lethality when the dominant allele is homozygous, which is similar to what has previously been reported by Hori and Hori *et al. *[14,19].

Previous studies have documented similar patterns of inheritance for jaw handedness in several groups of fishes [19,20] consistent with the hypothesis that craniofacial laterality is regulated, at least in part, by an evolutionarily conserved locus. We reasoned that, if the evolution of craniofacial asymmetries in the Perissodini were the consequence of selection upon a latent and conserved locus for jaw handedness, it would also segregate in other East African cichlids. Taking advantage of a preexisting cross between two herbivorous Lake Malawi species, we looked for a genetic signature of handedness in different oral traits. Indices of asymmetry were generated for various jaw dimensions and we found significant variation in the index of asymmetry for the length of the retroarticular process of the lower jaw among F_2 _hybrids. Handedness in this trait has been used previously to describe jaw laterality in other cichlid species [19]. We next performed a mapping experiment using laterality (left versus right) as our trait of interest and found a narrow region on linkage group 10 that was significantly associated with handedness of the retroarticular process of the lower jaw: markers significantly associated with the direction of retroarticular asymmetry were GM294 and UNH2101 (Figure 5E).

In order to evaluate whether or not this locus segregates with handedness in *P. microlepis*, larval samples from a single family were typed as either lefty or righty and genotyped at both microsatellite loci. Only 93 (of 141) animals showed unambiguous jaw laterality. While individuals were invariant for GM294, they exhibited a size polymorphism for UNH2101. Two alleles where recovered from this family: 'A' - 150 bp, and 'B' - 158 bp. The mother was scored as heterozygous and, given that all three genotypic classes were recovered, we deduced that the sire was also heterozygous. Using three additional microsatellites, we found no evidence for multiple paternity. A departure from expected genotypic frequencies was determined using a *X*^2 ^goodness of fit test for each morphotype and for the family as a whole. Association analysis showed a clear difference in genotypic frequencies among morphs with righties tending to be homozygous for the 'B' allele (*X*^2 ^= 32.89, df = 2, *P *< 0.0001) and lefties exhibiting a disproportionate number of heterozygous animals (*X*^2 ^= 14.13, df = 2, *P *= 0.0009). These data are consistent with breeding experiments that have demonstrated dominance of the lefty allele over the righty [19]. Notably, we also observed a deficiency in the frequency of 'A' homozygous animals, which is also consistent with previously documented patterns of inheritance in *P. microlepis*, specifically, early lethality of animals homozygous for the dominant allele [14,19]. In fact, patterns of inheritance at UNH2101 were not different from what would be expected under a model of a lefty (A/B) *x *lefty (A/B) cross with the dominant allele being homozygous lethal (*X*^2 ^= 3.49, df = 1, *P *= 0.062). The few A/A individuals in our family appeared normal and were lefty or righty with equal frequency.

## Conclusions

The evolution of lepidophagy in the Perissodini is marked by specific shifts in ecomorphology and an elaboration of craniofacial asymmetries. While asymmetries are evident throughout the genus *Perissodus*, their progression from simple sided differences in jaw size/length to more complicated, functionally significant, differences in jaw shape illustrates how crucial this adaption has been in the exploitation of scales as food source. Our developmental and genetic data strongly support a genetic basis for jaw laterality in cichlids but also suggest a degree of complexity in the morphogenesis of this trait that has not been previously recognized. We speculate that jaw laterality in adult *Perissodus *is the result of multiple 'layers' of asymmetric processes acting throughout ontogeny, including early patterning mechanisms, growth and remodelling and, potentially, behaviour. Notably, zebrafish lacking two functional copies of *fgf8 *exhibit jaw asymmetries that are strikingly similar to those in Lake Tanganyika scale eaters [28]. While *fgf8 *is not in the interval that segregates with jaw laterality in cichlids, it is still possible that it participates in the development of this trait as a member of a larger signalling network. Alternately, there could be several ways in which to lateralize the skull. Continued work in both laboratory models and natural populations should facilitate a better understanding of the genes and signalling networks involved in the lateralization of the vertebrate body plan.

## Methods

### Morphometric analysis

Seven of the nine species in the Perissodini tribe were included in this analysis [*Perissodus straeleni *(*n *= 9), *Perissodus microlepis*(*n *= 9), *Perissodus paradoxus*(*n *= 5), *Perissodus elaviae*(*n *= 2), *Perissodus multidentatus*(*n *= 2), *Perissodus hecqui*(*n *= 5) and *Haplotaxodon microlepis*(*n *= 4)]. Given that many Perissodini species are rare, and that our morphometric method was somewhat destructive, we were only able to procure individuals from a subset of species. However, good phylogenetic coverage is obtained with these seven species (Figure 2A). Specimens came from the personal collection of RCA at Syracuse University, the University of Michigan at Ann Arbor, Cornell University and the Royal Museum for Central Africa in Belgium. The left and right sides of each specimen's skull were dissected in order to remove skin and connective tissues, exposing a set of 16 landmarks, points that characterize the mechanics and geometry of the skull (Figure 1). Dissected specimens were photographed using an Olympus SP-570 and landmarks were digitized using the program tpsDig [32].

Lefty and righty individuals were grouped in this analysis and tests of laterality focused on the differences between sides of the skull that would be facing (towards) or opposite (away) the prey. The sides of each individual were characterized as either 'towards' or 'away' depending on whether, when photographed, the specimen's mouth was bending 'towards' or 'away' from the camera. The mandibular symphysis for all *Perissodus *species was within 5° (or 4% of total head length) of the sagittal midline of the skull. The degree of error due to the bending out of the plan of focus of the camera should, therefore, be minimal. Using tpsRelw [33], RW analysis was performed as previously described [28] in order to identify major axes of shape variation among all samples. The set of landmarks and methods used to analyse the skull of Perissodini species were adapted from previously published work on damselfish craniofacial morphology [34].

MA was calculated for jaw opening and closing in four *Perissodus *species as the ratio of in-lever to out-lever lengths [25]. In order to ensure the accurate planar orientation of the left and right sides of the jaw when photographed, we only used cleared and stained specimens for this analysis. Specimens of *P. straeleni*(*n *= 8), *P. microlepis *(*n *= 5), *P. paradoxus*(*n *= 6), and *P. hecqui *(*n *= 4) were enzymatically cleared with trypsin and bones were stained with Alizarin red using a method described by Potthoff [35]. These were the only species that we were able to obtain in sufficient numbers for this analysis but they provide good phylogenetic and ecological coverage (for example, deep- versus shallow-water predators) across the Perissodini. Photographs were taken of each side of the lower-jaw. Landmarks, pivot points and lever arms of the lower-jaw were placed using tpsDig [32] and MAs were calculated. Analyses of variances were used to test for asymmetries in MAs within species.

### Larval *P. microlepis*

A brood of 141 *P. microlepis *was collected from the mouth of a wild-caught female and staged to the late larval period following [26]. The posterior half of each animal was taken for DNA isolation and genotypic analysis, and the anterior half was cleared and stained according to the method described by Walker and Kimmel [36] in order to visualize bone and cartilages. Skeleton preparations were photographed in the dorsal and ventral views using a Zeiss Axiocam digital imaging system mounted to an M2 Bio stereomicroscope (Zeiss) and processed used Adobe Photoshop CS4.

Our method for quantifying asymmetric mouth direction in these samples followed that of Hori *et al. *[19]. It involved measuring and comparing the angles formed by the lines between the left and right preorbital processes and from the preorbital processes to the symphysis of the upper jaw. A measure of laterality for each specimen was obtained by taking the difference between the left and right angles (L-R as in [19]).

### Linkage analyses

A hybrid cross, that was originally generated in order to identify loci that segregate with jaw shape [37,38], was used to determine whether or not the handedness of the oral jaw is genetically determined in Lake Malawi cichlids. The F_2 _mapping population (*n *= 173) was generated by crossing two Lake Malawi cichlid species, *Labeotropheus fuelleborni *and *Metriaclima zebra*. We used a linkage map that assigned 165 markers (both microsatellites and SNPs) to 25 linkage groups using JoinMap 3.0 [39]. Details of the map construction have been published elsewhere [37,38]. We used the nonparametric mapping function of MapQTL 4.0 [40] to estimate the genomic position of jaw handedness. This approach employs the Kruskal-Wallis rank sum test and is described in detail in a paper by Streelman *et al.*[41].

### Marker association analysis in wild *P. microlepis *larvae

Of the 141 *P. microlepis *larvae, 93 could be reliably scored as either lefty or righty. A subset (*n *= 25) of these animals was genotyped at microsatellite loci linked to handedness. Primers were GM294F/R GCTCGTCCTATCTTTAGAACA/AAACCAGCCCGCTATT, and UNH2101F/R CTGCAGGGTCAAGTTTTCGT/GGCTGGGAGGAGAAAGAAAT. GM294 was found to be invariant but UNH2101 showed a size polymorphism. The remaining larvae were grouped by handedness and genotyped at UNH2101. A *X*^2 ^goodness of fit test was used to test for a departure from expected genotypic frequencies for each morphotype, and for the family as a whole.

## Abbreviations

MA: mechanical advantage; RW: relative warp.

## Authors' contributions

RCA designed the experiments. TAS performed the morphometric experiments. TAS and RCA performed the genetic mapping and association analysis. Both authors analysed and interpreted the results and wrote the manuscript.
